# Matching Your Face or Appraising the Situation: Two Paths to Emotional Contagion

**DOI:** 10.3389/fpsyg.2017.02278

**Published:** 2018-01-04

**Authors:** Huan Deng, Ping Hu

**Affiliations:** ^1^School of Education, Huaibei Normal University, Huaibei, China; ^2^Department of Psychology, Renmin University of China, Beijing, China

**Keywords:** emotional contagion, emotional mimicry, social appraisal, zygomaticus major (ZM), corrugator supercilii (CS)

## Abstract

Emotions are believed to converge both through emotional mimicry and social appraisal. The present study compared contagion of anger and happiness. In Experiment 1, participants viewed dynamic angry and happy faces, with facial electromyography recorded from the zygomaticus major and corrugator supercilii as emotional mimicry. Self-reported emotional experiences were analyzed as emotional contagion. Experiment 2 manipulated social appraisal as the gaze of expression toward the target. The results showed that there was emotional contagion for angry and happy expressions both in Experiment 1 and Experiment 2. Experiment 1 indicated an overt mimicry pattern for happy faces, but not for angry faces. Experiment 2 found an influence of social appraisal on angry contagion but not on happy diffusion. The two experiments suggest that the underlying processes of emotional mimicry and social appraisal are differentially relevant for different emotional contagion, with happiness processing following a mimicry-based path to emotional contagion, and anger processing requiring social appraisal.

## Introduction

Emotions are believed to converge through social networking. A person can acquire emotions, such as anger and happiness, from people around him or her. This process is called emotional contagion, whereby emotional expression of a person leads another person to experience a congruent emotional state (Bruder et al., 2012; Peters and Kashima, 2015). Researchers have considered the two most influential accounts of emotional contagion: emotional mimicry and social appraisal. The emotional mimicry account holds that the emotional expression of others may be automatically mimicked (Chartrand and Bargh, 1999), thus via autonomous feedback processes, leading directly to convergence in subjective feelings (Hatfield et al., 1993). According to the social appraisal theory, the emotional expression of others confers important information about the situation, and we may come to a congruent appraisal of the situation and congruent emotion with others (Manstead and Fischer, 2001; Bruder et al., 2014). To our notion, theoretical thinking is ripe enough to explore different processes of emotional contagion in empirical research. After an overview of research on emotional mimicry and social appraisal, we present two studies testing their influences on emotional contagion.

Emotional mimicry is defined as the imitation of the facial, verbal, or postural expressions of others (Hatfield et al., 1993; Hess and Fischer, 2013). Newborn babies will cry when they hear others crying; the corners of our mouths will unconsciously rise when seeing others' smile. Dimberg et al. (Dimberg, 1982; Dimberg and Söderkvist, 2011; Dimberg and Thunberg, 2012) measured emotional mimicry through emotion-specific facial electromyographic (EMG) activities. The zygomaticus major (ZM), which increases while smiling, is measured as happy mimicry, and activity of the corrugator supercilii (CS) to angry faces is used to assess frowning. According to the facial feedback hypothesis, the facial muscles function as a feedback system for a person's own experience of emotion (Strack et al., 1988). Hatfield et al. (1993) further illustrated the relationship between emotional mimicry and emotional contagion, wherein by automatically mimicking the facial expression of others, the subjective emotional experience of the receiver is affected by the feedback from facial muscles. Evidences for mimicry-based emotional contagion have been provided by studies reporting emotion-specific EMG responses of participants to angry and happy faces (Dimberg, 1990; Dimberg et al., 2000), and emotional changes of participants when their muscles are measured or manipulated (Strack et al., 1988; Dimberg and Söderkvist, 2011; Dimberg and Thunberg, 2012; Sato et al., 2013). To study the neural association between emotional mimicry and emotional contagion, Hennenlotter et al. (2009) applied botulinum toxin (BTX) to the corrugator supercilii muscle to inhibit angry mimicry. Participants viewed emotional faces during functional magnetic resonance imaging scanning. The results showed that, compared to control group, the BTX group exhibited impaired brow lowering and reduced activation of the left amygdala during imitation of angry expressions, suggesting the influence of emotional mimicry on emotional contagion.

Though there is evidence of mimicry-based emotional contagion, some studies did not confirm the direct relationship between emotional mimicry and emotional contagion (Hess and Blairy, 2001; Tamietto et al., 2009). Some researchers hold that emotional mimicry is only common at birth; and is replaced by some advanced cognitive processes later, such as social appraisal (Hoffman, 2002). Social appraisal is the process of re-appraising the situation with information from the emotion of others. Thus, the “behaviors, thoughts, or feelings of one or more other persons in the emotional situation are appraised in addition to the appraisal of the event *per se*”(Manstead and Fischer, 2001). Objects with a happy face were more liked than those with a disgusted face (Bayliss et al., 2007); recognition of emotion was improved when the contextual emotional expressions gazed toward the target face (Mumenthaler and Sander, 2012, 2015). The change in situational appraisal can thus result in similar emotional state. Parkinson and Simons (2009) found that in naturalistic settings, anxiety and excitement of another person could affect reported emotions of participants via their risk and importance appraisals. Additionally, a highly fearful friend could infer risky situation and induce similar panic (Lawrence-Wood, 2011).

Given to the strong evidences for each path, several studies have compared mimicry-based emotional contagion and appraisal-based emotional contagion in one study. Some researchers found a role of emotional mimicry and social appraisal for one kind of emotional contagion (Parkinson and Simons, 2009; Hawk et al., 2011), while others are more inclined to support the appraisal-based contagion (Lawrence-Wood, 2011; Bruder et al., 2012). However, to ensure ecological validity, these studies are not carried out in a more experimental environment, which can constantly measure emotional mimicry, social appraisal, and emotional contagion. Moreover, most researchers studied only one kind of emotional contagion, not comparing emotions of different characteristics, such as positive and negative emotions. Different routes of emotional contagion are sensitive to different situations (Peters and Kashima, 2015), so these routes may also apply to different emotions. Thus, the present study compared contagion of anger and happiness in controlled experiments, while measuring the degree of contagion, emotional mimicry, and manipulating social appraisal.

In Experiment 1, we followed the standard procedure for studying emotional mimicry and emotional contagion to compare the mimicry-based paths of dynamic angry and happy faces. Though prevalent, emotional mimicry tends to differ among different emotions. People are more likely to get in touch with others via mimicking happy expression rather than angry one (Seibt et al., 2015). Hence, the mimicry-based contagion of happiness may be more intense than that of anger. Then in Experiment 2, we manipulated social appraisal as the gaze of expression (Mumenthaler and Sander, 2012), to compare the appraisal-based contagion of anger and happiness, wherein emotional mimicry was also measured. Anger, being complicated and often related to surroundings (Berkowitz and Harmon-Jones, 2004), in order to be diffused, should require more information from the expressions of others. Thus, we predicted that anger diffusion should be more influenced by social appraisal, while mimicry-based contagion of happiness may still exist.

## Experiment 1

### Method

#### Participants

Thirty undergraduate students (24 female, 6 male; mean age = 20.8 years, SD = 2.54) from Renmin University of China participated in the experiment to fulfill a course requirement. They had normal or corrected to normal eyesight and none of them reported neurological diseases. After explaining the experiment, we obtained their informed written consents. The study was approved by the Institutional Review Board of the Department of Psychology at Renmin University of China.

#### Stimuli

**Figure 1:**
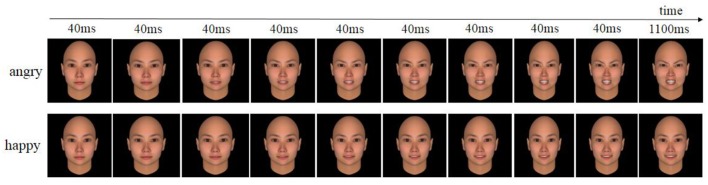
Example of the pictures used for an angry and a happy stimulus.

Twenty neutral faces (10 females and 10 males) were selected from the Chinese Facial Affective Picture System (CFAPS) (Wang and Luo, 2005). To control the intensity of emotional expressions, we synthesized dynamic angry or happy expressions of the same face identity using FACSGen (software developed by the Swiss Center of Affective Sciences; see Krumhuber et al., 2012), gradually increasing emotional intensity (0, 15, 30, 45, 60, 70, 80, 90, 100, and 110% intensity) for each emotion and each identity, identical to a previous procedure (Achaibou et al., 2008). Prior test showed that both happy and angry dynamic expressions were highly recognized, with the accuracy of 85.0 and 89.3%, respectively. We presented the picture sets one after the other with the Eprime software (Psychology Software Tools, http://www.pstnet.com). In each set, the first nine pictures lasted for 40 ms, and the last one was presented for 1,100 ms, creating dynamic angry or happy expressions (see Figure 1 to see an example of the pictures used). In total, 40 different picture sets were created following this procedure.

#### Procedure

Following a standard procedure (Achaibou et al., 2008), the subjects were individually tested while seated in a comfortable chair in a sound-attenuated laboratory room. After the EMG electrodes were placed, they were exposed to 40 facial clips (20 angry, 20 happy). Each clip (1,460 ms duration) was preceded by a central fixation cross for 1,000 ms, and separated by a varying intertrial interval (800–1,000 ms). The order of presentation of movie clips was randomized for each subject. Participants passively viewed the movie clips, and rated their experienced emotion (i.e., the type and strength of emotion felt by participants upon perceiving the expression), using a 9-point scale (1 = very happy, 4 = a little happy, 5 = neutral, 6 = a little angry, 9 = very angry) after each clip. The subject was told that the sweat gland activity in their faces was being measured, masking the real purpose.

#### Apparatus and data analysis

The EMG activity of each muscle was measured using the Biopac system EMG (BIOPAC Systems, Inc., Santa Barbara, CA), with a high-pass frequency filter. Facial EMG was measured by surface Ag/AgCl bipolar electrodes placed over the ZM and the CS on the left side of the face, according to the guidelines given by Fridlund and Cacioppo (1986). A ground electrode was placed on the left mastoid. The EMG was continuously recorded at 2,048 Hz, with a 20–500 Hz bandpass filter.

Subsequently, EMG data were analyzed using AcqKnowledge software version 3.5 (Biopac Systems). The raw data were transferred into EMG signals by calculating the root-mean-square (RMS) every 100 ms. The data were segmented into 2,460 ms epochs, including 1,000 ms of prestimulus baseline and 1,460 ms of stimulus exposure for each trial. EMG responses were scored by subtracting baseline from each 1,460 ms interval. Trials in which the EMG response was superior to 3 standard deviations of the overall mean value were rejected. A mean EMG response-from-baseline waveform was finally obtained for each subject and condition.

To confirm the existence of emotional contagion, the rating data were averaged over each emotion, and were analyzed with repetitive measure analysis of variance (ANOVA), together with a test for a difference from a five (neutral experience) using one-sample *t*-tests. Additionally, to determine if specific facial muscle activity corresponded to the expected patterns of mimicry, EMG data were analyzed as a function of muscle (two levels: ZM vs. CS) and emotion (2 levels: angry vs. happy). To further investigate the time course of EMG activity, comparisons between values at each time-bin (14 levels: 100 ms time-bins from 0 to +1,400 ms post-stimulus onset) for the response to angry and happy stimuli were assessed by paired *t*-tests. Finally, to directly test the relationship between emotional mimicry and emotional contagion, correlations were calculated.

### Results

#### Emotional contagion

To assess emotional contagion, repetitive measure analysis of variance (ANOVA) was conducted on the self-reported experiences. Happy faces induced more happiness (M ± SD, 3.632 ± 0.827), while angry faces induced more anger (7.197±1.000), *F*_(1, 29)_ = 150.270, *p* < 0.001, η^2^ = 0.838. Moreover, self-reported experiences both differed from neutral experience [anger, *t*_(29)_ = 12.026, *p* < 0.001; happiness, *t*_(29)_ = −9.054, *p* < 0.001], indicating successful emotional contagion.

#### EMG data

**Figure 2:**
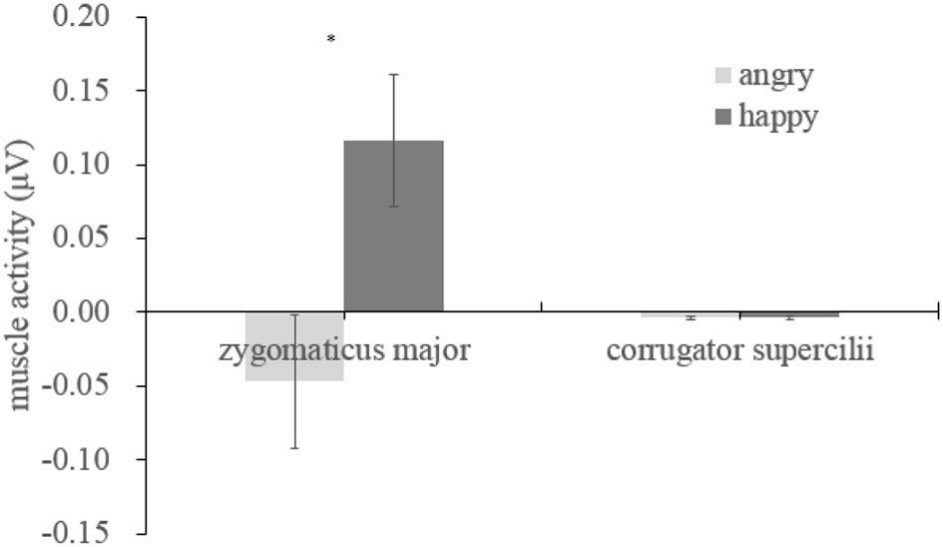
Mean activity (μV) for angry and happy expressions, ^*^p < 0.05.

We examined the EMG activity at the ZM and CS in response to angry and happy faces (see Table 1) compared to the proceeding baseline. As indicated by Figure 2, there were significant interactions between muscle and emotion [*F*_(1, 29)_ = 7.534, *p* = 0.01, η^2^ = 0.206], suggesting different activity for the two muscles as a function of the facial expression. A subsequent simple effect analysis showed that the ZM was more activated by happy than by angry faces [*F*_(1, 29)_ = 7.490, *p* = 0.01], which was not apparent in the CS [*F*_(1, 29)_ = 0.01, *p* = 0.92], indicating an overt mimicry pattern for happy expression, but not for angry faces.

**Table 1:** EMG activity in ZM and CS in response to angry and happy faces (M ± SD).

**ZM**		**CS**	
**Angry**	**Happy**	**Angry**	**Happy**
−0.047 ± 0.247	0.116 ± 0.245	−0.004 ± 0.004	−0.004 ± 0.004

**Figure 3:**
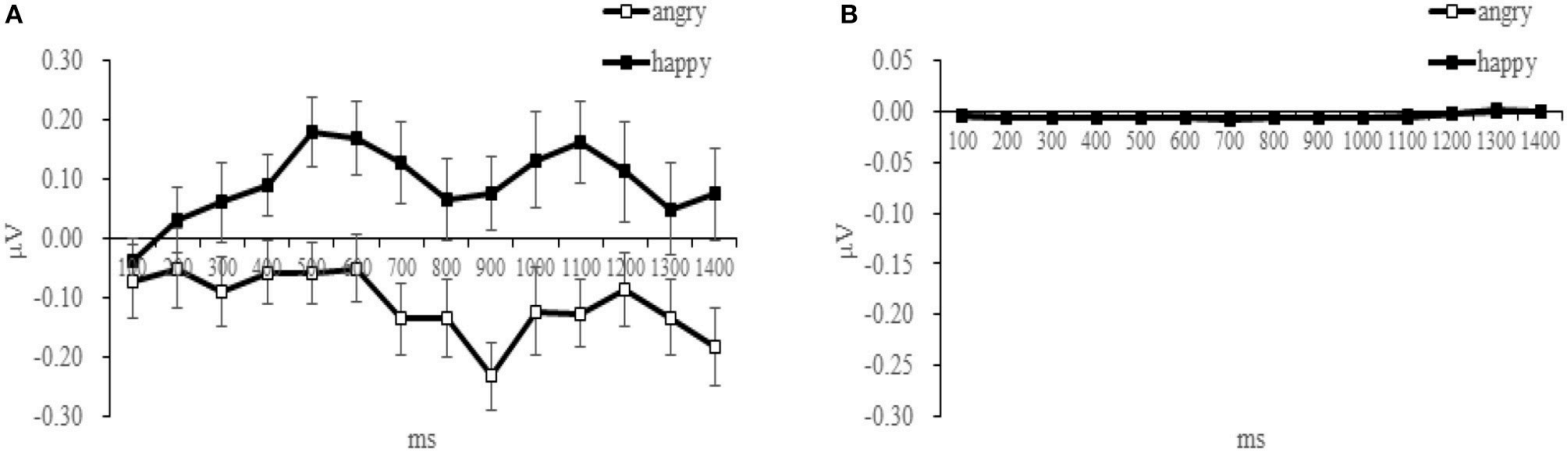
Mean activity (μV) for angry and happy expressions recorded in the ZM **(A)** and in the CS **(B)**, plotted in 100 ms bins after the stimulus onset.

The time-course of muscular response to both facial expressions is shown in Table 2 and Figure 3 for both muscles. Paired *t*-tests at each successive time-bin showed that the EMG responses to these two expressions was significantly different from 400 ms onwards for the ZM muscle [*t*_(14)_ = −2.293, *p* = 0.029], but not different for the ZM muscle, demonstrating a differential time-course of activation for these two muscles (Table 2 provides a more detailed statistical descriptions). As illustrated in Figure 3, the ZM response was larger in response to happy facial expressions than to angry facial expressions, whereas their difference was not seen for the CS.

**Table 2:** (a) Zygomaticus major and (b) corrugator supercilii activity in response to angry and happy faces for each time interval followed by the statistics [Significant *p*-values (*p* < 0.05) are in bold].

	**Time interval in ms**													
	**100**	**200**	**300**	**400**	**500**	**600**	**700**	**800**	**900**	**1,000**	**1,100**	**1,200**	**1,300**	**1,400**
**(a) ZYGOMATICUS MAJOR**														
Angry (μV)	−0.073	−0.050	−0.090	−0.058	−0.059	−0.051	−0.136	−0.134	−0.233	−0.124	−0.126	−0.085	−0.133	−0.183
Happy (μV)	−0.036	0.032	0.061	0.090	0.181	0.169	0.129	0.067	0.077	0.133	0.162	0.114	0.050	0.075
*T*-value	−0.491	−1.035	−1.771	−2.293	−3.450	−2.586	−3.034	−2.357	−4.092	−2.781	−3.444	−2.759	−2.621	−3.265
*p*-value	0.627	0.309	0.087	**0.029**	**0.002**	**0.015**	**0.005**	**0.025**	**0.000**	**0.009**	**0.002**	**0.010**	**0.014**	**0.003**
**(b) CORRUGATOR SUPERCILII**														
Angry (μV)	−0.003	−0.006	−0.006	−0.006	−0.006	−0.006	−0.007	−0.006	−0.005	−0.005	−0.004	−0.001	0.001	0.000
Happy (μV)	−0.003	−0.005	−0.005	−0.006	−0.006	−0.005	−0.006	−0.006	−0.005	−0.005	−0.005	−0.001	0.002	0.001
*T*-value	−0.162	−0.724	−0.814	−0.125	0.290	−0.626	−0.272	−0.258	0.072	0.025	0.220	−0.022	−0.896	−0.261
*p*-value	0.872	0.475	0.422	0.901	0.774	0.536	0.788	0.799	0.943	0.980	0.827	0.982	0.377	0.796

Due to the co-existence of contagion and mimicry for happy faces, we calculated the correlations between self-reported experience and ZM activity in response to happy expressions. However, they were not significantly correlated, *r*_(28)_ = −0.027, *p* = 0.889, which did not directly support mimicry-based emotional contagion.

### Discussion

In Experiment 1, we measured the emotional contagion and emotional mimicry in response to angry and happy dynamic faces. As predicted, there exists emotional contagion of angry and happy expressions. However, they differed in emotional mimicry, with happy faces evoking larger zygomaticus muscle activity; and angry faces without larger corrugator muscle activity, which suggested different paths of contagion.

In previous studies, happy mimicry was stable, while angry mimicry emerged now and then. According to the social contextual view of emotional mimicry (Hess and Fischer, 2013), mimicry of emotion signals social bonds and may be impaired when there exist negative consequences. Angry faces often imply something threating or antagonistic. The mimicry of happiness is therefore more likely than that of anger. In addition, in some cultures, anger is more regulated by the social norms, which may also explain the lack of angry mimicry (Rymarczyk et al., 2016).

In contrast to angry faces, the co-existence of mimicry and contagion in response to happy faces implies mimicry-based contagion of happiness. However, we did not find a direct relationship, which replicated previous studies (Blairy et al., 1999). The result is not strong enough to exclude the possibility of mimicry-based contagion. Among current studies exploring the relationship between emotional mimicry and contagion, emotional contagion is mostly manipulated as self-reported emotional experience, which varies less than EMG activity (emotional experience to happy faces, 3.632 ± 0.827; ZM to happy faces, 0.116 ± 0.245). Emotional mimicry could be one way of sharing emotions, but this pattern should be more obvious between motor cortexes and affective brain areas, which represent emotional mimicry and emotional contagion, respectively (Bastiaansen et al., 2009).

In Experiment 1, we found different patterns of emotional mimicry between angry and happy contagion that provided more support for mimicry-based contagion of happiness, though we still knew nothing about angry contagion. Thus, in Experiment 2, we introduced another path of emotional contagion, social appraisal, which may be more sensitive to angry contagion.

## Experiment 2

### Method

#### Participants

Thirty-eight undergraduate students (26 female, 12 male; mean age = 20.6 years, SD = 2.79) from Renmin University of China participated in the experiment to fulfill a course requirement. As in Experiment 1, they had normal or corrected to normal eyesight and none of them reported neurological diseases. After explaining the experiment, we also obtained their informed written consents. The study was also approved by the Institutional Review Board of Department of Psychology at Renmin University of China.

#### Stimuli

Twenty neutral faces (10 females and 10 males) were selected from the Chinese Facial Affective Picture System (CFAPS; Wang and Luo, 2005). Emotional expressions (angry, happy) and gaze directions (left, right) were manipulated by FACSGen (Krumhuber et al., 2012). To create realistic dynamic emotional expressions, angry and happy facial expressions of inter-mediate intensity were created, resulting in 80 sets of dynamic frames (neutral-direct gaze, neutral-left or right gaze, 50% emotion-left or right gaze, 100% emotion-left or right gaze).

Target stimuli that showed two persons interacting were photographed. Adobe Photoshop (Adobe Systems Incorporated, San Jose, CA) was used to blur the target stimulus, in order to create an uncertain situation that could induce both angry and happy emotions.

#### Procedure

We created our procedure using previously described methods for assessing gaze-directed orienting (Fichtenholtz et al., 2007) and social appraisal (Mumenthaler and Sander, 2012; Soussignan et al., 2015). The subjects were individually tested while seated in a comfortable chair in a sound-attenuated laboratory room. After the EMG electrodes were placed, they performed the 4 experimental conditions: 2 (context condition: social appraisal and non-social appraisal) × 2 (emotion: angry and happy). Each condition consisted of 20 trials for a total of 80 trials per participant, with a short break to avoid fatigue.

**Figure 4:**
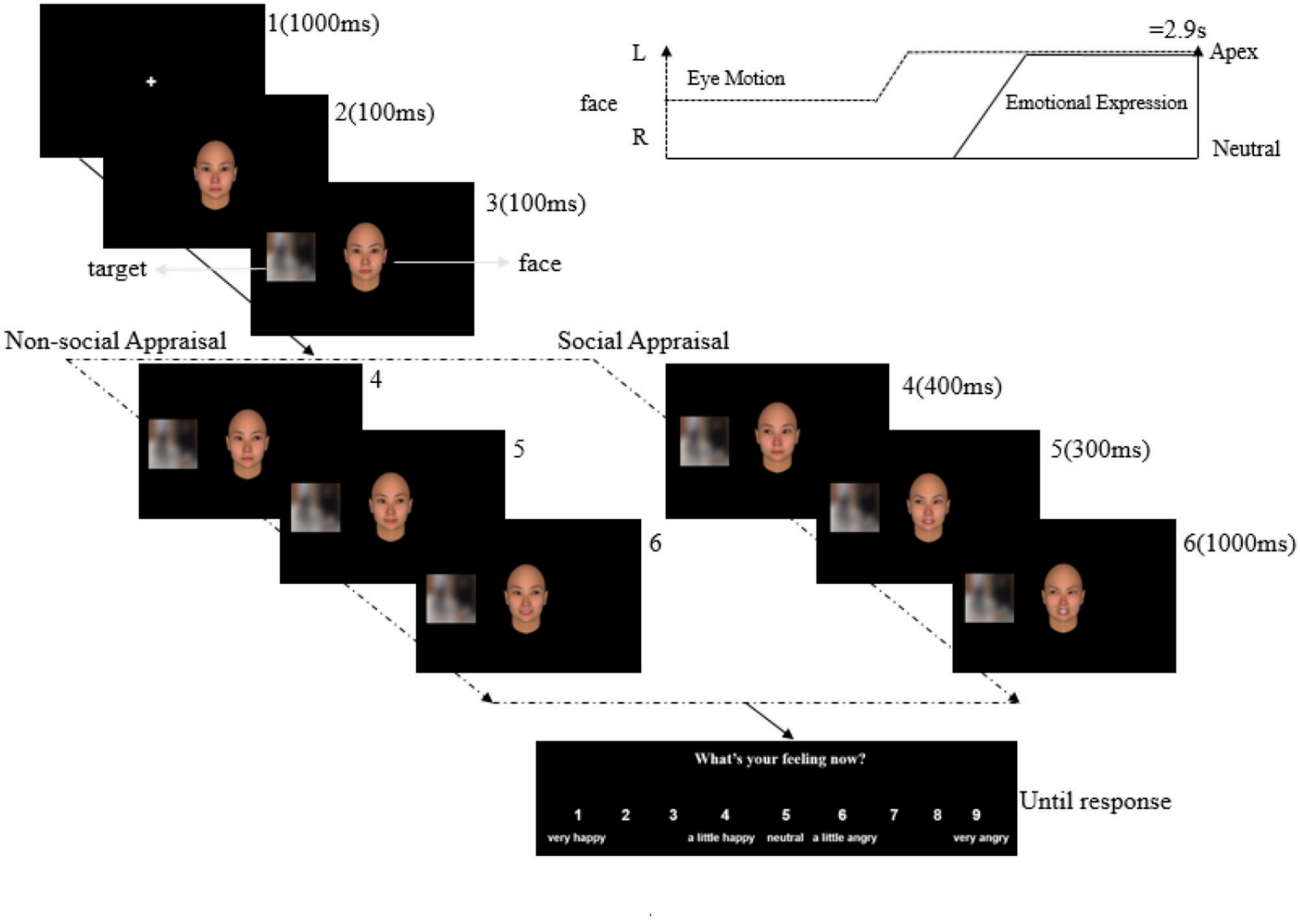
Illustration of an experimental trial for Experiment 2. After the presentation of the fixation cross (1), the face appeared in the center of the screen (2), followed by the target in the periphery of the screen (3). The gaze of the face shifted (4) away from the target (non-social appraisal condition) or toward the target (social appraisal condition). Following the gaze shift, the face expressed 50% emotion (5), and then expressed 100% emotion (6).

For each trial, a neutral face with a straight gaze appeared after a 1,000-ms fixation cross, which remained for 100 ms. Then, the target appeared on the left or right side of the face for another 100 ms, with the face still in the center of the screen. To create the condition of social appraisal, the face showed a gaze shift (400 ms) toward the target. In non-social appraisal condition, the gaze was directed away from the target. Following the gaze shift of the face, it displayed a 50% angry or 50% happy expression (300 ms), then turned to a 100% angry or 100% happy expression for 1,000 ms. The total duration of the dynamic sequence in all conditions was 2.9 s (1,000 ms for the fixation cross; see Figure 4). Then participants were asked to rate their experienced emotion using a 9-point scale (1 = very happy, 4 = a little happy, 5 = neutral, 6 = a little angry, 9 = very angry) in the following response window.

#### Apparatus and data analysis

The EMG activity of each muscle was measured and analyzed the same way as Experiment 1, except for the epoch time. EMG data were segmented into 2,700 ms epochs, including 1,000 ms of prestimulus baseline and 1,700 ms of stimulus exposure after the gaze shift of the face.

The rating data were averaged over each condition, and were analyzed as a function of emotion (2 levels: angry, happy) and context (two levels: social appraisal, non-social appraisal) in repeated-measures ANOVAs. EMG data were also analyzed as a function of muscle (two levels: ZM, CS), emotion (2 levels: angry, happy) and context (two levels: social appraisal, non-social appraisal).

### Results

#### Emotional contagion

**Figure 5:**
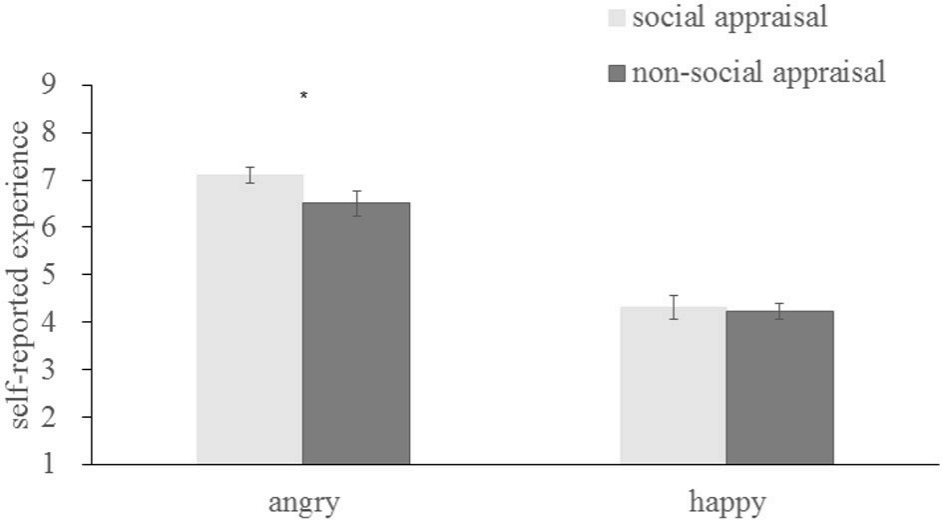
Self-reported experience for angry and happy expressions, ^*^p < 0.05.

To assess emotional contagion, self-reported experiences were analyzed as a function of emotion and context. There was a significant main effect of emotion [*F*_(1, 37)_ = 53.183, *p* < 0.001, η^2^ = 0.59], with angry faces inducing more anger (6.804 ± 1.193), happy faces inducing more happiness (4.269 ± 1.1379), and both different from neutral [anger, *t*_(37)_ = 9.323, *p* < 0.001; happiness, *t*_(37)_ = −3.960, *p* < 0.001]indicating successful emotional contagion. There was also a significant interaction between emotion and context [*F*_(1, 37)_ = 11.246, *p* = 0.002, η^2^ = 0.233]. As indicated in Figure 5, a subsequent simple effect analysis showed that, angry faces in the social appraisal condition (7.106 ± 1.052) induced more anger than those in the non-social appraisal condition (6.502 ± 1.645) [*F*_(1, 37)_ = 7.17, *p* = 0.011], while happy faces in both condition were not significantly different (social appraisal, 4.313 ± 1.553; non-social appraisal, 4.225 ± 1.072) [*F*_(1, 37)_ = 0.15, *p* = 0.697], which implied the influence of social appraisal for angry contagion but not for happy diffusion.

#### EMG data

**Figure 6:**
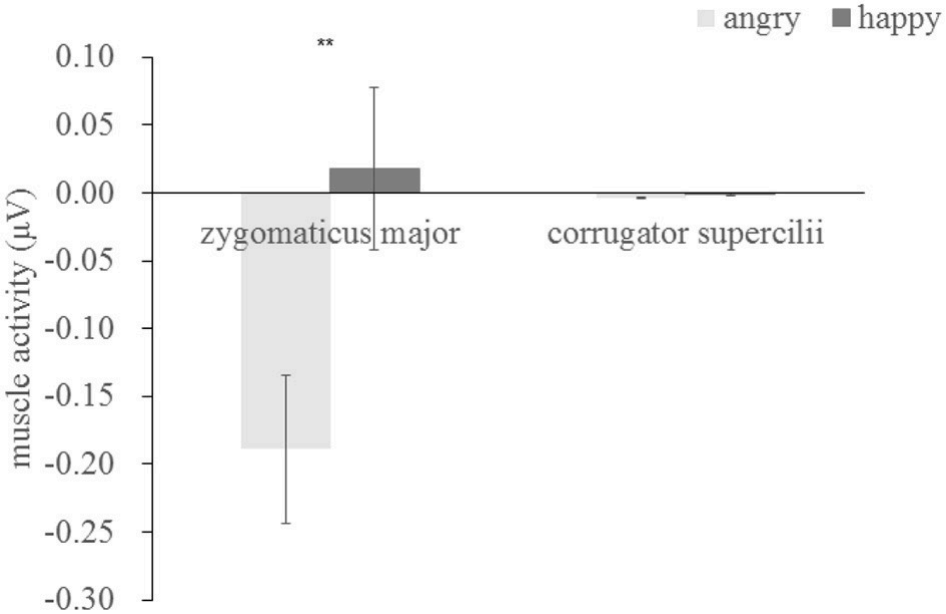
Mean activity (μV) for angry and happy expressions, ^**^p < 0.01.

Similar to Experiment 1, we examined EMG activity as a function of muscle, emotion and context. The three-way repeated-measures ANOVA showed a significant main effect of emotion [*F*_(1, 37)_ = 8.51, *p* = 0.006, η^2^ = 0.187], and a significant interaction between muscle and emotion [*F*_(1, 37)_ = 8.133, *p* = 0.007, η^2^ = 0.18]. Other main effects and interactions were not significant. As indicated by Figure 6, a subsequent simple effect analysis showed that the ZM was more activated by happy than by angry faces [*F*_(1, 37)_ = 11.67, *p* = 0.002], which was not parent in CS [*F*_(1, 37)_ = 0.10, *p* = 0.775], indicating an overt mimicry pattern for happy expression, but not for angry faces (see Table 3 for summary of EMG activity).

**Table 3:** EMG activity (μV) in ZM and CS in response to angry and happy faces (M ± SD).

**ZM**		**CS**	
**Angry**	**Happy**	**Angry**	**Happy**
−0.189 ± 0.335	0.018 ± 0.370	−0.004 ± 0.004	−0.001 ± 0.006

Due to the co-existence of emotional mimicry in both experiments, we ran a mixed- ANOVA to compare Experiment 1 and Experiment 2 (averaged across social appraisal and non-social appraisal versions). The EMG means for ZM were analyzed in ANOVA with group (Experiment 1, Experiment 2) as between-subjects factor and emotion as within-subjects factor. The mixed-ANOVA showed a significant main effect of emotion [*F*_(1, 66)_ = 14.678, *p* < 0.001, η^2^ = 0.182], a significant main effect of group [*F*_(1, 66)_ = 4.21, *p* = 0.044, η^2^ = 0.060]. And the interaction between emotion and group was not significant, [*F*_(1, 66)_ = 0.206, *p* = 0.652, η^2^ = 0.003]. These replicated results in Experiment 1 and Experiment 2, indicating mimicry for happy expressions. Meanwhile, ZM activity in Experiment 1 was stronger than that in Experiment 2, suggesting a greater degree of happy mimicry.

Due to the co-existence of mimicry and experience convergence for happy faces, we also calculated the correlations between ZM activity and self-reported experience in response to happy expressions. However, identical to Experiment 1, they were not significantly correlated, *r*_(36)_ = −0.166, *p* = 0.319, which did not directly support mimicry-based happy contagion.

### Discussion

On the basis of Experiment 1, we manipulated social appraisal as the gaze of faces toward the target, while non-social appraisal as the gaze away from the target in Experiment 2. As predicted, both anger and happiness were successfully converged. However, they differed in the mechanism, with angry contagion affected by social appraisal and happy contagion coexisting with emotional mimicry.

The gaze of other is amazing, it can shift our attention toward the observed object, and we can impose motor and emotive components of others into the object via gaze (Becchio et al., 2008). In the social appraisal condition, emotional contagion was affected by the interaction between the gaze, which enriches the target of the meaning of the expression, and the emotional expression of the face (Mumenthaler and Sander, 2012, 2015). Thus, the difference between gaze directions should signal the effect of social appraisal with the mere contextual effect excluded. In our study, angry faces in the social appraisal condition induced more anger than those in the non-social appraisal condition, while happy faces in both conditions were not significantly different, which implied the influence of social appraisal for angry contagion but not for happy diffusion. We interpreted this result based on the characteristics of anger. Anger often arises when some external thing is seen as the cause of the offense, and the reverse feeling could result in aggressive reactions toward the offense (Berkowitz and Harmon-Jones, 2004). Thus, complex, time-consuming references about the situation may be necessary before we respond to the anger of others.

## General discussion

Emotions are social. This fact means that how we feel is affected by the emotions of others. The present research was designed to investigate how the emotions of others influence our own emotional reactions. The findings of the two experiments reported here generally provide support for the hypothesis that individuals tend to emotionally converge with others. When others expressed happiness, participants reported more happy feelings, together with a tendency for emotional mimicry (both in Experiment 1 and Experiment 2). In contrast, when others expressed anger, more angry feelings were reported, as a function of social appraisal (in Experiment 2). Thus, the two experiments give some support for the prediction that the primary path for emotional contagion differs among different emotions, with happiness following a mimicry-based path, and anger processing requiring social appraisal.

Emotions have evolved with different adaptive values. It is evolutionarily adaptive to be more sensitive to bad things than to good ones (Tay, 2015). Thus, compared to happiness, which has rapid and effortless response, we attend to angry faces with more attention and cognitive resources to figure out and fight against the potential threats. This evolutionary advantage may explain why angry contagion uses more energy-consuming social appraisal than happy contagion.

Though this research initially compared the converging mechanism of anger and happiness, and found some interesting differences between them, thus enriching the study of emotional contagion, there are still some directions for future research. First, in our study, we failed to find direct evidence of mimicry-based happy contagion. As discussed above, emotional mimicry varies more sensitively, and future research can improve the measurement of emotional contagion, using either the degree of emotion change or a neural index, to find some direct relationship between emotional mimicry and emotional contagion. In addition, in order to manipulate social appraisal in Experiment 2, we used an ambiguous picture as the target, which may promote appraisal-based contagion of anger. Under conditions of low uncertainty, there will be some automatic influence of both emotional mimicry and social appraisal in emotional contagion, and social appraisal processes are likely to be much more influential when uncertainty is high (Bruder et al., 2014). It is interesting for future studies to decrease situational uncertainty to unearth the synergy between emotional mimicry and social appraisal.

## Author contributions

HD and PH: designed the study; HD: performed data collection and analysis; HD and PH: wrote the paper; All authors approved the final version of the paper for submission.

### Conflict of interest statement

The authors declare that the research was conducted in the absence of any commercial or financial relationships that could be construed as a potential conflict of interest.
